# Factors associated with sexual disorders in women followed for breast cancer

**DOI:** 10.1192/j.eurpsy.2023.1354

**Published:** 2023-07-19

**Authors:** F. Zaouali, I. Abbes, A. Ben Slama, I. Belhaj Youssef

**Affiliations:** 1 Outpatient Medical Oncology consultation, Haj Ali Soua regional hospital, Ksar Hellal, Monastir; 2 Family Medicine Department, University of Monastir, Monastir; 3 Outpatient Physical Medicine and Rehabilitation consultation, Haj Ali Soua regional hospital, Ksar Hellal, Monastir, Tunisia

## Abstract

**Introduction:**

Breast cancer is the most frequently encountered malignant tumor among women in Tunisia and in the world. The quality of sexual life of patients with breast cancer is impaired by multifactorial mechanisms.

**Objectives:**

The aim of our study was to determine the factors associated with sexual disorders in patients followed for breast cancer.

**Methods:**

Cross-sectional analytic study including patients followed for breast cancer at the outpatient medical oncology consultation of Hadj Ali Soua regional hospital from January to March 2021. We collected sociodemographic and clinical data with an assessment of sexuality (FSFI), marital satisfaction (MAT), psychological profile (HAD) and quality of life (SF36).

**Results:**

Fifteen patients were included with a mean age of 49.87 ± 8.48 years and a mean age at diagnosis of 46.73 ± 7.55 years. At the TNM classification, 66.6% of the patients had a T1 or T2 at the time of diagnosis and 80% had an N0. All patients received a surgical intervention, which was conservative in 53.3% of cases. No patient underwent breast reconstruction. Chemotherapy and hormone therapy were prescribed in 86.7% of patients. The mean score of the FSFI questionnaire in our study was 17.25. Eleven patients (73.3%) had an FSFI score below 26.55. We found negative correlations between age and FSFI score (r=-0.622; p=0.013). We noted statistically significant negative correlations between FSFI and HAD-D (r=-0.606; p=0.017) and FSFI and HAD-a (r=-0.707; p=0.01) as well as significant correlations between FSFI and the following items: RE (r=0.84p=0.000), SF (r=0.684 p=0.005), GH (r=0.671 p=0.006) and MCS (r=0.788 p=0.000).

**Conclusions:**

Focusing on a small sample of patients followed for breast cancer, our study provides an assessment of the sexual function in its various areas and shows how sexuality is deeply intertwined with other sections of medical management.

**Disclosure of Interest:**

None Declared

